# Extraction and characterization of bovine collagen Type V and its effects on cell behaviors

**DOI:** 10.1093/rb/rbac028

**Published:** 2022-05-23

**Authors:** Jun Xu, Xi Luo, Yang Zhang, Jianping Gao, Ching-Cheng Huang, Xinpeng Bai, Guifeng Zhang

**Affiliations:** College of Food Science and Engineering, Engineering Research Center of Utilization of Tropical Polysaccharide Resources, Ministry of Education, Hainan University, Haikou 570228, China; PARSD Biomedical Material Research Center (Changzhou), Changzhou 213176, China; State Key Laboratory of Biochemical Engineering, Institute of Process Engineering, CAS, Beijing 100190, China; State Key Laboratory of Biochemical Engineering, Institute of Process Engineering, CAS, Beijing 100190, China; State Key Laboratory of Biochemical Engineering, Institute of Process Engineering, CAS, Beijing 100190, China; PARSD Biomedical Material Research Center (Changzhou), Changzhou 213176, China; Department of Biomedical Engineering, Ming-Chuan University, 32033 Taiwan, China; College of Food Science and Engineering, Engineering Research Center of Utilization of Tropical Polysaccharide Resources, Ministry of Education, Hainan University, Haikou 570228, China; PARSD Biomedical Material Research Center (Changzhou), Changzhou 213176, China; State Key Laboratory of Biochemical Engineering, Institute of Process Engineering, CAS, Beijing 100190, China

**Keywords:** collagen Type V, bovine cornea, fiber diameter, cell migration

## Abstract

Collagen Type V (Col. V) plays an essential role in cell behaviors and has attracted increasing attention in recent years. High-purity Col. V is needed for evaluating its biological properties. In this research, the enzymatic hydrolysis process was combined with ultrafiltration to purify Col. V from the bovine cornea. The purity of Col. V was determined to be above 90% by both sodium dodecyl sulfate-polyacrylamide gel electrophoresis (SDS-PAGE) and high-performance liquid chromatography methods. The effect of Col. V on cell behaviors was evaluated. The circular dichroism spectroscopy results demonstrated that the extracted Col. V exhibited a complete triple helix structure. SDS-PAGE suggested that the molecular weight of Col. V was 440 kDa. The self-assembly experiment revealed that the proportion of Col. V in the collagen mixture can affect the Col. I fiber diameter. The cell culture results implied that Col. V can inhibit fibroblasts (L929) proliferation. The L929 showed maximum mobility when the addition of Col. V was 30%. Thus, Col. V has the effect of inhibiting L929 proliferation and promoting migration. The high-purity Col. V provides useful information for further understanding its biological implications.

## Graphical Abstract

**Figure:**
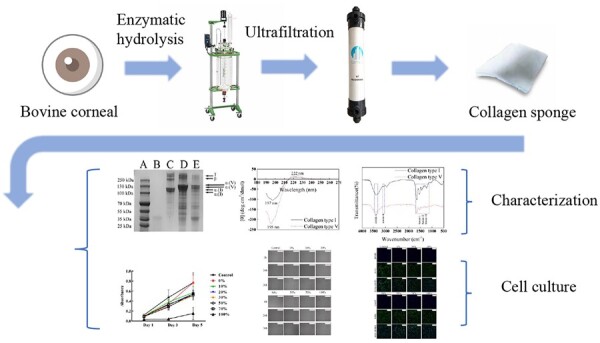

## Introduction

Collagen is the main extracellular protein in the connective tissue, accounting for 30% of the total protein of vertebrates [1]. The ubiquity of collagen in tissues has promoted its use in medical devices tailored for a broad range of applications. Different types of collagen present various bioactivities such as protein–protein interaction, regulation of cell behaviors and tissue properties.

Collagen Type V (Col. V) is a quantitatively minor fibrillar collagen. Three varieties of chains exist in Col. V, including α-1, α-2 and α-3, and each structure contains two propeptides, namely, P5NP (N-terminal) and P5CP (C-terminal). The N-terminal propeptides consist of signal peptides, a thrombospondin domain or von Willebrand factor C domain depending on the chain and a noncollagenous domain. Col. V can combine with DNA, heparan sulfate, thrombospondin, heparin and insulin. It can control the initiation of collagen fibril assembly and regulate the diameter of Col. I and Col. III assembly [2, 3]. Col. V is essential for fibrillation of Col. I [4]. It is commonly discovered in various connective tissues, such as skin, cornea, skeletal muscle and placenta [5]. Col. V, which is a growth regulator of pluripotent islet organs, can inhibit bone differentiation of mesenchymal stem cells [6, 7]. Col. V influences the fibrillary formation and tissue quality and contributes to the bone matrix, corneal stroma and the interstitial matrix of muscles. There are many forms of Col. V in different tissues, with [α_1_(**V**)]_2_[α_2_(**V**)] as the main form. The composition of the propeptide of Col. V in each tissue is different. The abnormal content or the ratio of chain composition is associated with many diseases, such as classic Ehlers-Danlos syndrome, pulmonary fibrosis and myocardial infarction [8, 9]. Therefore, it is imperative to extract high-purity Col. V for scientific research.

The common methods for collagen extraction include salt precipitation, alkali or acid extraction, and enzymatic method [10]. Collagen exhibits low thermal stability and high concentration salts. Besides, the denaturation of collagen will be caused by alkali extraction. The acid-based method coupled with enzymatic hydrolysis presents advantages over the salts or alkali-based method. Different types of collagens possess similar molecular weight (MW) and isoelectric points. It is difficult to separate Col. V from the other collagens with high abundance. Many methods for extracting Col. V have been reported, while the collagen purity was not provided, and the structure and physiological function of Col. V was not characterized in detail [11]. Thus, it is of great significance to develop a novel method for the separation of Col. V with high purity.

In this study, a collagen extraction process was investigated. Col. V makes up 10–20% of the fibril-forming collagens in the corneal stroma [12]. It was adopted as the raw materials for collagen extraction based on a combination of acid-enzymatic hydrolysis and membrane separation. The extracted Col. V was characterized by sodium dodecyl sulfate-polyacrylamide gel electrophoresis (SDS-PAGE), Fourier transform infrared spectroscopy (FTIR), UV spectrum, circular dichroism (CD), differential scanning calorimetry (DSC), scanning electron microscopy (SEM) and transmission electron microscopes (TEM). The property of regulation of Col. V on collagen fiber diameter was explored to reveal its effects on cell proliferation, migration and morphology.

## Materials and methods

### Materials

Bovine corneas were purchased from the local market. Hollow fiber ultrafiltration membranes (Beijing Xubang Membrane Equipment Co., Ltd., China), porcine pepsin (SIGMA, USA), bovine Col. V ELISA (Shanghai Yanzun Biotechnology Co., Ltd., China). Col. I standard (National Institutes for Food and Drug Control, China), high MW marker (Thermo, USA), mouse fibroblasts (L929 cell lines) (Beijing Cell Bank of the Chinese Academy of Science, China), cell counting kit-8 (Dojindo, Japan), DAPI (Thermo, USA), actin-tracker green (Shanghai Biyuntian Biotechnology Co., Ltd., China). All the reagents used were of analytical grade.

### Extraction process

The Col. V extraction was modified according to a widely used method which has been reported by Wu, and the original Wu’s Col. V extraction method was used as the control [11, 13]. The modified method was divided into three steps: (i) enzymatic hydrolysis, (ii) salt precipitation with low pH and (iii) purification.

Enzymatic hydrolysis: the fresh cornea was washed with 1 mol/l NaCl solution containing 2 mol/l urea, rinsed with deionized water, and then homogenized. The homogenized cornea was suspended in 0.5 mol/l acetic acids, with the addition of pepsin solution (1%, w/w). Following the volume of pepsin solution, the ratio of total protein in the cornea to pepsin was 100:1. The hydrolysis process was conducted at 4°C for 24 h. The reaction mixture was filtered using a mesh, and the filtrate was collected. The suspension was homogenized again and added to the filtrate. The suspension was kept at 4°C for 48 h. The enzymatic hydrolysate was centrifuged at 10 000 rpm for 15 min, and the supernatant was collected.

Salt precipitation with low pH: salt precipitation process was used to separate Col. V contained in the supernatant. NaCl powder was gradually added to the supernatant to a final concentration of 0.7 mol/l. The solution was kept at 4°C for 12 h and then centrifuged at 10 000 rpm for 30 min. Afterward, Types I and V of the supernatant containing collagen were collected while Types I and IV of the precipitate containing collagen were discarded.

Purification: the supernatant was ultrafiltered using a hollow fiber ultrafilter with an MW cut-off of 100 kDa under 0.08 kPa. Subsequently, the retentate was poured into a dialysis bag with MW cut-off of 100 kDa dialyzing against deionized water for 24 h, during which the water was changed every 4 h. The collagen solution was collected and further purified using neutral salt precipitation. NaCl was added to the solution. The concentrations were kept at 1.0 mol/l for 24 h. Then, the salt concentration was increased to 2.4 mol/l and maintained at 4.0 mol/l for 24 h. Next, the solution was centrifuged at 10 000 rpm for 10 min to remove insoluble precipitates. The supernatant was dialyzed again with the similar process mentioned above. The solution in the bag was freeze-dried, and the sample is Col. V.

### Characterization of Col. V

#### Purity of Col. V

The purity of Col. V was indicated by the determination of the collagen content in the sample. The Col. V extracted by the proposed method and the control method were quantified with bovine Col. V ELISA kit, respectively. The collagen solution with a concentration of 1 mg/mL was prepared by dissolving the extracted collagen sample in 1 mM HCl. Later, nine volumes of the collagen solution in 1 mM HCl were mixed with one volume of 200 mM phosphate buffer (PB) containing 1.5 M NaCl, pH 7.3, at 4°C. The mixture was diluted with 200 mM PB to an appropriate concentration, and the content of Col. V was determined with an ELISA kit. ELISA experiments were performed using Spectra Max M5 multi-function microplate reader (Molecular Devices, USA).

#### SDS-PAGE analysis

SDS-PAGE was conducted following the literature [12]. The Col. I standard and MW marker were employed to characterize the MW of extracted Col. V. The impurity proteins and other types of collagen contained in Col. V were extracted by the proposed method and the control methods were evaluated by the SDS-PAGE.

#### Amino acid analysis

The amino acid composition was determined based on Yuswan *et al.* [14]. The Col. V sample and Col. I standard were hydrolyzed in the gaseous phase with 6 M HCl at 110°C for 16 h. The contents of Hyp and collagen from various tissues were detected as reported in the reference [15]. The liberated amino acids were derived with DNFB and analyzed by high-performance liquid chromatography (HPLC) on a Pico Tag 4.6 mm × 250 mm column (Agilent, USA). According to the peak area of the sample and the standard, the amino acid concentration in the sample was calculated, then the number of amino acid defects was calculated according to the amino acid concentration, and finally, the amino acid composition was obtained.

#### FTIR spectrum

The samples and KBr were mixed in a mortar with a mass ratio of about 1:100. Then, the Col. V sample and Col. I were pressed into a uniform transparent sheet with a hydraulic instrument. The light transmittance of the biofilm and the Col. I were measured at a wavelength of 400-4000 cm^−1^ with a NICOLET iS 50 spectrophotometer (Thermo, USA), with a scanning resolution and a scanning time of 4 cm^−1^ and 100 s, respectively.

#### UV spectrum

The UV absorption spectra of collagen were recorded with a spectrophotometer. The Col. V sample and Col. I standard were dissolved in 0.5 mol/l acetic acids to prepare a collagen solution with a concentration of 0.5 g/L. Next, the collagen was scanned in the near-ultraviolet light region of 200-400 nm by an ultraviolet-visible spectrophotometer to obtain the ultraviolet Maximum absorption wavelength.

#### CD spectrum

CD spectrum was recorded using Jasco J-810 Spectropolarimeter (Jasco, Japan). The Col. V sample and Col. I standard were dissolved in 0.5 mol/l acetic acids to prepare a collagen solution with a concentration of 0.5 g/L. The samples were centrifuged at 10 000 rpm for 10 min. The collagen solution was loaded in a quartz cell of 1 mm path length. The wavelength scanning was performed in the range of 190-260 nm with a scan rate of 50 nm/min at 25°C. The same sample was measured repeatedly 3 times, with 0.5 mol/l acetic acids as a blank control.

#### DSC analysis

The Col. V sample and Col. I standard were dissolved in 0.5 mol/l acetic acids to prepare a collagen solution with a concentration of 0.5 g/L. The samples were centrifuged at 10 000 rpm for 10 min. After being degassed for 30 minutes, the temperature of samples were raised from 25°C to 85°C at a heating rate of 1°C/min to determine the heat distortion temperature with DSC (GE, USA).

#### SEM and TEM analysis

The lyophilized collagen samples were sputter-coated with gold. The surface morphology and structural characteristics were examined using a JSM-6700F field emission scanning electron microscope (Jasco, Japan) at different magnifications.

The collagen samples were dissolved in 0.5 mol/l acetic acids to prepare a solution with a concentration of 1 mg/mL. The samples were dialyzed with phosphate buffer (200 mmol/L, pH7.4) at 4°C for 48 h. The microstructure of the collagen fibrils formed in the different conditions was observed with an electron JSM-6700F microscopy (Jasco, Japan) at an acceleration voltage of 15 kV. After all samples were incubated at 37°C for 60 min, the collagen gel was fixed in tin foil with 2.5% (v/v) formalin in phosphate buffer for 12 h and dehydrated in ethanol with stepwise concentrations of 0%, 10%, 20%, 30%, 50%, 70%, 90%, and 100% (v/v) for 15 min. Fibril diameters of SEM images were measured, and the mean of 100 fibrils was reported in each case.

TEM samples were prepared by placing the collagen suspensions on copper grids with 200 mesh size and removing excess water by placing a piece of filter paper at the edge of the grid. Afterward, the fibrils were negatively stained with 1% phosphotungstic acid at pH 7.4 for 15 s and then air-dried. D-periodicities were measured with EM-1400Flash (Jeol, Japan,). The mean of 50 D-periodicities was reported in each case. Statistical analysis software was Image J V 1.30.

### Biocompatibility properties

#### Cell proliferation

The collagen samples were dissolved in 0.5 mol/l acetic acids to obtain a 1 mg/mL solution and dialyzed against phosphate buffer (200 mmol/L, pH 7.4) at 4°C for 2 days. The collagen samples were prepared into solutions of different concentrations of 5 µg/mL, 10 µg/mL, 50 µg/mL, 100 µg/mL, and 500 µg/mL with phosphate buffer (200 mmol/L, pH 7.4). Then, the samples were immediately distributed to 96-well tissue culture plates; 50 µL per well was added to each group of 6 wells; they were incubated at 37°C for 60 min to form a collagen gel. Subsequently, the collagen solution was aspirated, and the well plate was sterilized under ultraviolet light for 30 minutes. The sterilized collagen samples were hydrated with 1640 medium for 12 h, and 1640 medium was carefully removed before cell inoculation. Besides, L929 were cultured in a humidified incubator containing 5% CO_2_ at 37°C for three days. The growth of the cells was observed with an inverted microscope, and the optimal collagen concentration for cell growth was selected for follow-up experiments.

The Col. V sample and Col. I standard were mixed in different proportions, as exhibited in Table 1. Then, the collagen samples were divided into 96-well tissue culture plates; 6 wells were added to each group; 50 µL of the sample was added to each well, with 6 wells as one group. The samples were placed at 37°C for 1 h to form a gel. Subsequently, the collagen solution was removed, and the well plate was sterilized under ultraviolet light for 30 min. The sterilized collagen sample was hydrated with 1640 medium for 12 h, and the 1640 medium was carefully removed before cell inoculation. Next, 1 × 10^3^ cells/mL of cells were seeded onto the wells of a 96-well plate and incubated for 72 h in an air atmosphere with 5% CO_2_. The viability of cultured L929 was monitored using the colorimetric CCK-8 assay after 72 h of culture, and the optical density (OD) was measured at 450 nm using a microplate reader. No substance was added to the control group except the culture medium, and 10% dimethyl sulfoxide was added to the blank group.

**Table 1.** The proportion of collagen configuration

Column	A	B	C	D	E	F	G
Col. I	100	90	80	70	50	30	0
Col. V	0	10	20	30	50	70	100

#### Cell scratch test

According to the proportion in Table 1, the Col. V sample and Col. I standard were mixed. Then, 100 µL of the mixed collagen samples were pipetted into each well of a 6-well tissue culture plate. The samples were placed at 37°C for 1 h to form a gel. Cells were seeded onto the wells at a density of 1 × 10^4^ per mL and then incubated for 72 h in an air atmosphere with 5% CO_2_. After the cell growth reaches 80% confluence, scratches of uniform width were produced with a 200 µL pipette tip. Adherent cells were scratched and rinsed 3 times with phosphate buffer solution. Subsequently, 1640 medium containing only 2% bovine serum was added. Cell growth was observed and photographed with an inverted phase-contrast microscope at 0 h, 12 h, 24 h, 36 h, 48 h, and 72 h after the scratch and a control group without collagen was conducted. The area of the cell scratch was measured by Image J V 1.30 image analysis software.

#### Cell morphology

Collagen was coated on a confocal dish using the cell scratch method and a control group without collagen coating was conducted. Cells were seeded onto the confocal dish at a density of 1 × 10^4^ per mL and incubated for 72 h in an air atmosphere with 5% CO_2_. The confocal dish adopted 4% paraformaldehyde fixative solution to fix cells overnight and rinsed cells for 3 × 5 min with 0.1% Triton X-100 in PBS. Additionally, 1 ml of actin-tracker green staining solution was diluted by adding 50 ml of PBS (including 1% bovine serum albumin and 0.1% Triton X-100). Each sample was added with 200 µL of actin-tracker green staining solution, incubated at room temperature for 20-60 min, and washed 2-3 times with 0.1% Triton X-100 in PBS. After the first step of dyeing was completed, 5 µL of DAPI staining solution was added to stain the small dish for 2 min and washed 2-3 times with PBS. Later, the confocal dish was observed and photographed using a confocal Leica SP8 STED 3X microscope (Leica, Germany).

## Results

### Purity of Col. V

**Figure 1.**
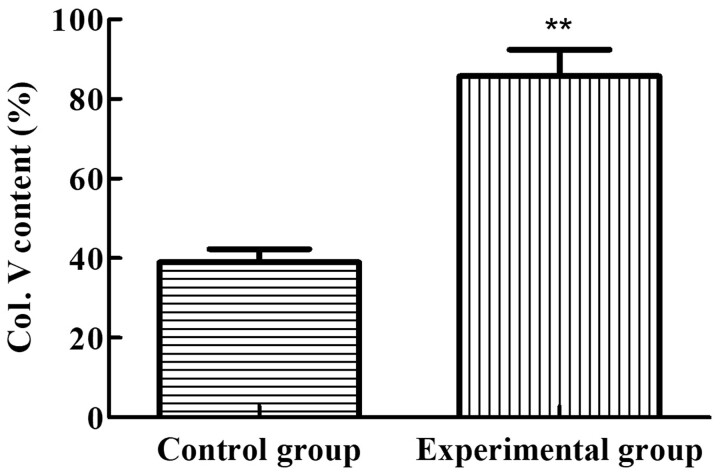
Purity of collagen Type V obtained by two methods. **P < 0.01 vs. control

Figure 1 shows that the purity of Col. V in the control group and the experimental group were 38.99 ± 4.66% and 85.67 ± 9.46%, respectively, with a high significance (**P < 0.01). According to these results, the method proposed in this study can obtain higher purity Col. V, which proves the superiority of this method.

#### SDS-PAGE analysis

A: MW marker; B: pepsin (10 μg); C: standard of bovine Col. I (10 μg); D: control group (10 μg); E: experimental group (10 μg).

**Figure 2.**
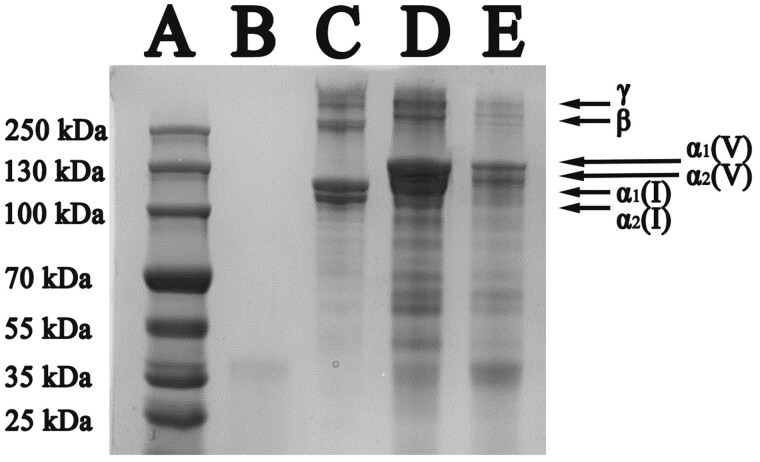
SDS-PAGE of Col. V extracted by two methods

The MW and impurity proteins of the extracted Col. V were determined by SDS-PAGE. As shown in lane C of Fig. 2, a band at 110 kDa and a band at120 kDa exist for Col. I standard, corresponding to α1 and α2 chains, respectively. Regarding Col. V extracted from the bovine cornea, two bands were observed, where the bond at 150 kDa is related to α1 chain of Col. V, and the bond at 140 kDa is associated with the α2 chain of Col. V. Since Col. V was composed of two α1 chains and one α2 chain, the MW of Col. V was calculated as 440 kDa. Compared with lane B, there were pepsin residues in both the experimental group (lane E) and the control group (lane D), and both the control group and the experimental group contained pepsin residuesIt can be clearly observed from lanes D and E that more Col. I and other impurity proteins were remained in the control group, while the Col. V obtained in the experimental group showed higher purity with less impurity proteins.

#### Amino acid analysis

The proportion of Hyp in mammalian collagen was about 12% [16]. Hyp was detected by HPLC to calculate the content of collagen in Col. V. The amino acid composition of Col. V is exhibited in Table 2. The Hyp content was 10.6%, based on which the total collagen in the sample was 91.27 ± 1.34%. The glycine in the sample was 326 per 1000 residues, accounting for 1/3 of total residues. Proline and hydroxyproline were 119 and 106 per 1000 residues, respectively. This results follows the typical composition of collagen.

**Table 2.** Amino acid composition of Col. I and Col. V

Amino acids	Col. I (residues/1000 residues)	Col. V (residues/1000 residues)
Asp	33	60
Glu	117	116
Hyp	101	106
Ser	29	25
Thr	9	53
Gly	311	326
Pro	119	119
Ala	112	41
Arg	55	49
Val	29	27
Met	0	0
Ile	13	13
Leu	24	28
Trp	0	0
Phe	5	7
His	10	12
Cys	0	0
Lys	30	14
Tyr	3	4
Total	1000	1000

#### FTIR analysis

**Figure 3.**
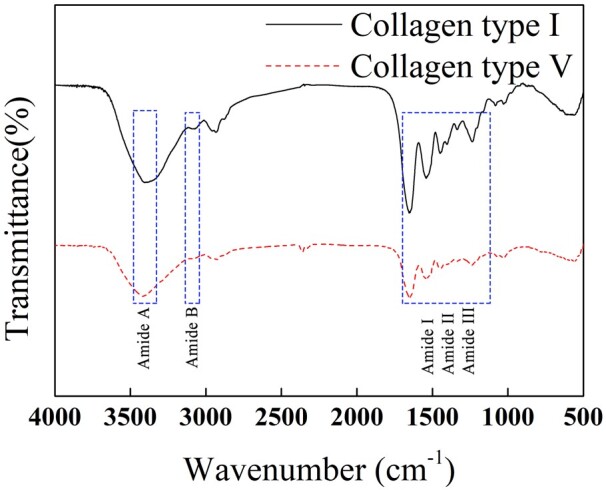
FTIR spectra of Col. I and Col. V

Figure 3 illustrates the FTIR analysis of Col. I and Col. V. The main characteristic absorption peaks are amide A, B, and amide I, II, III and IV bands [17]. Amide A band, 3400–3450 cm^−1^, is related to N–H stretching vibration or O–H stretching vibration of the hydrogen bond. The absorption peaks of Col. V amide A band of Col. I and Col. V are 3404.76 cm^−1^ and 3416.34 cm^−1^, respectively. The amide B band, 3080–3100 cm^−1^, is associated with the antisymmetric contraction vibration of the –CH_2_ group. The absorption peaks of the amide B band of Col. I and Col. V are 2933.72 cm^−1^ and 2933.24 cm^−1^, respectively. The amide I band, 1600–1660 cm^−1^, is connected with the C = O stretching shock. The absorption peaks of the amide I bands of Col. I and Col. V are 1654.17 cm^−1^ and 1653.687 cm^−1^, respectively. This region sensitively reflects the protein secondary structure. Amide II band, 1500–1600 cm^−1^, is correlated with N–H bending vibration and C–H stretching vibration. The absorption peaks of the amide I band of Col. I and Col. V are 1540.87 cm^−1^ and 1541.352 cm^−1^, respectively. The amide III band corresponds to various side-chain groups. The absorption peaks of the amide III bands of Col. I and Col. V are 1236.167 cm^−1^ and 1239.542 cm^−1^, respectively. Col. V extracted by the proposed acid enzyme method presents a complete triple helix structure, consistent with the characteristic absorption of collagen.

#### UV analysis

**Figure 4.**
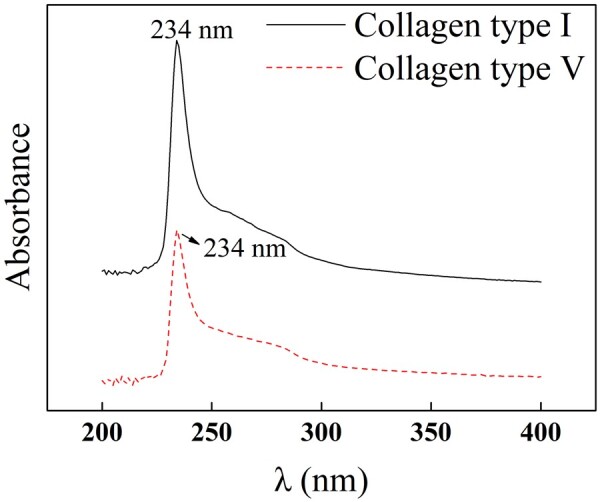
UV spectrum of Col. I and Col. V

Collagen usually has strong UV characteristic absorption with absorbance peaks around 220–230 nm and exhibits no characteristic absorption at 280 nm [18]. Figure 4 indicates that the maximum absorption peaks of Col. I and Col. V are both 234 nm, which is related to collagen hydroxyproline and proline. This observation is consistent with literature reports and conforms to the characteristic absorption of collagen [19].

#### CD spectrum analysis

**Figure 5.**
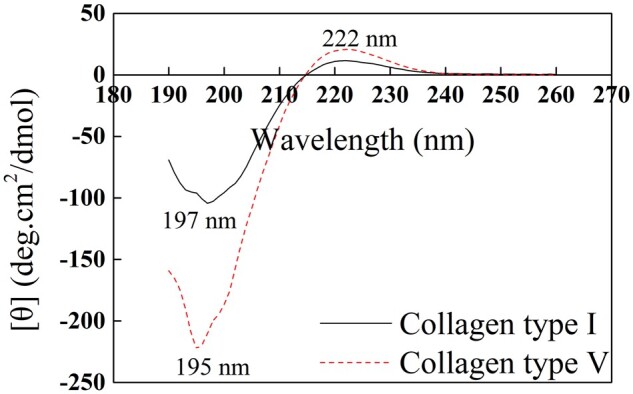
CD spectrum of Col. I and Col. V

Figure 5 displays the CD spectrum of Col. V. The maximum positive peak at 220–222 nm, a negative peak at 195–200 nm and a crossover point at 213 nm were observed [20]. On complete denaturation, the positive peak of collagen at 220 nm disappeared completely, the negative band has red-shifted, and partially denatured collagen exhibited lower intensity [21]. Besides, the ratio of the positive peak to the negative peak (RPN) is unique to the triple helix structure and can be adopted to identify the triple helix structure. The collagen RPN value ranges from 0.09 to 0.15. Figure 5 suggests that the largest positive peaks of Col. I and Col. V are at 222 nm, and the largest negative peaks are at 197 nm and 195 nm, respectively. Meanwhile, the RPN values of Col. I and Col. V are 0.112 and 0.093, respectively. The CD spectrum reveals that the obtained Col. V keeps the triple helix structure.

#### DSC analysis

**Figure 6.**
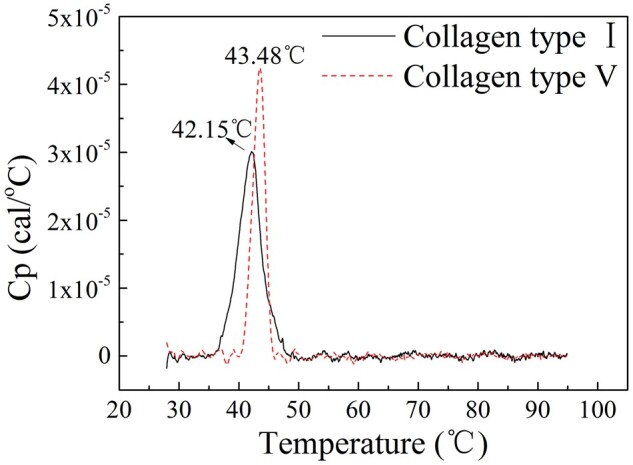
DSC thermograms of Col. I and Col. V

Figure 6 illustrates that the thermal denaturation temperature of Col. V is 43.48°C, which is higher than that of Col. I, 42.15°C. This is consistent with the results reported in the reference [22].

#### Morphological analysis

**Figure 7.**
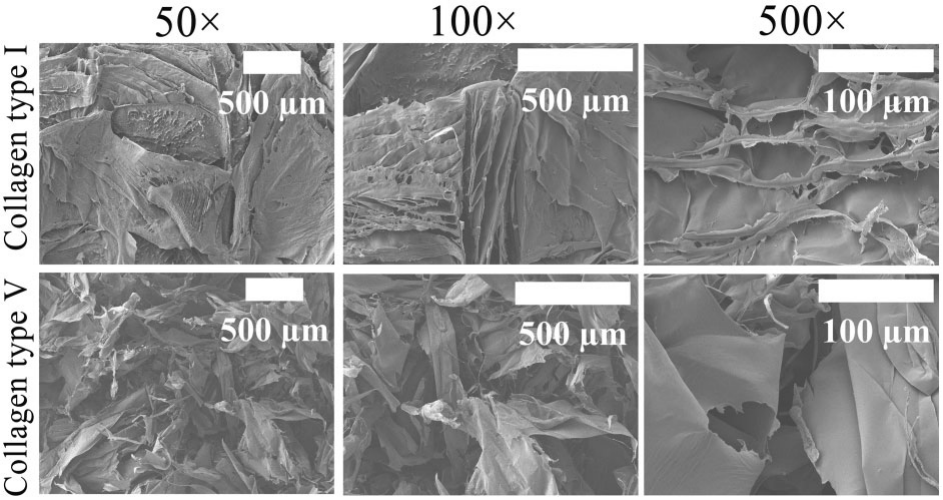
SEM micrographs of Col. I and Col. V

Figure 7 exhibits that the sponge of Col. I and Col. V under the same condition has different pore sizes, surface areas and porosities. The surface of Col. I was slightly smoother than that of Col. V. The rough surface may increase the surface area for the solid sample. For the collagen-based solid samples, the porosity and fibrillary structure would enhance cell adherence and proliferation. Generally, the homogeneous pore structure provides a suitable environment for the adhesion, proliferation, migration of cells and formation of new tissues [12]. Differences in morphology may have different biological effects. The porosity and fibrillar structure of the extracted Col. V exhibited a promising ability to act as a prominent substrate for cell adherence and proliferation.

#### SEM and TEM analysis

**Figure 8.**
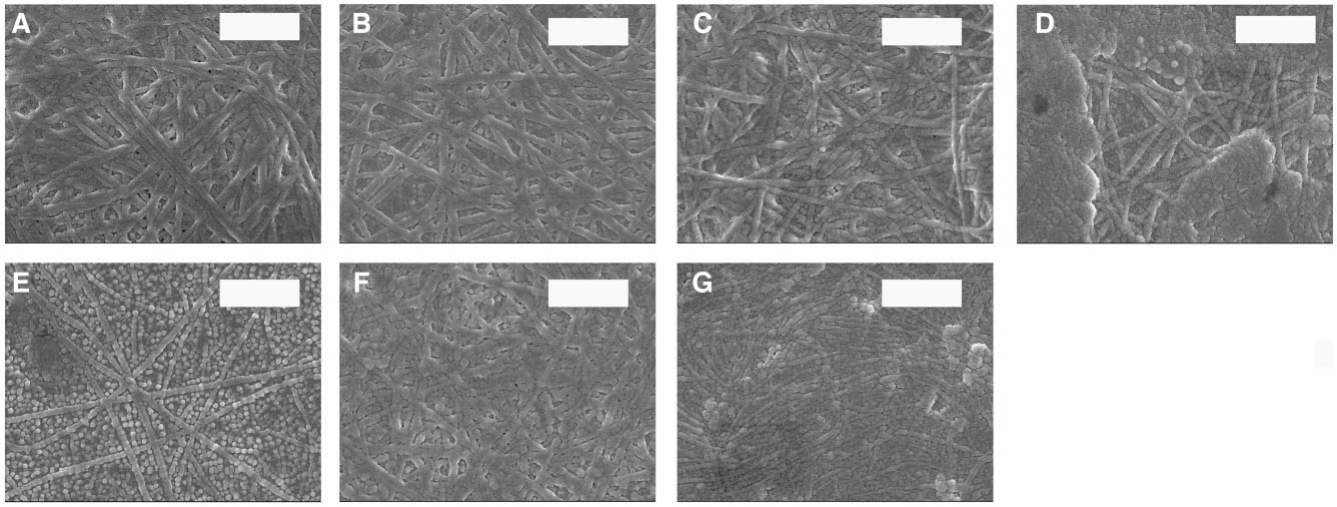
SEM of co-assembly of Col. I and Col. V. Scale bar: 1000 nm. The total concentration of collagen is 50 µg/mL, and (**A–G**) is the amount of Col. V added (A: 0, B: 10%, C: 20%, D: 30%, E: 50%, F: 70%, G: 100%)

**Figure 9.**
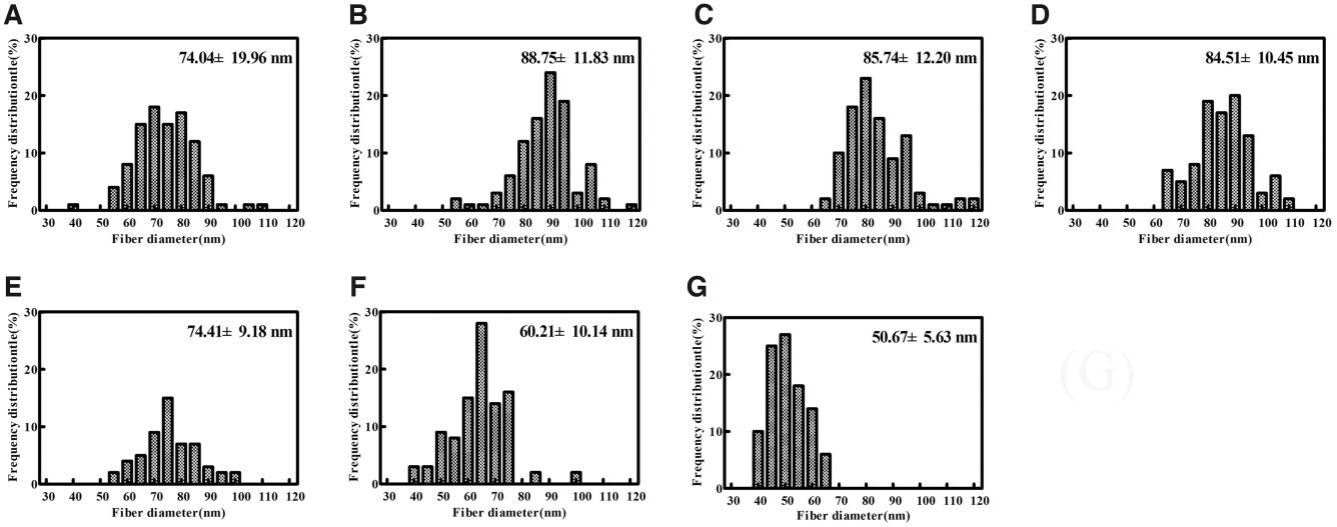
Fibril diameter of co-assembly of Col. I and Col. V. The total concentration of collagen is 50 µg/mL, and (**A–G**) is the amount of Col. V added (A: 0, B: 10%, C: 20%, D: 30%, E: 50%, F: 70%, G: 100%)

Figures 8 and 9 present the SEM of co-assembly of Col. I and Col. V. With an increase in the Col. V proportion, the average diameter first increases and then decreases. Col. I formed a broad distribution of relatively large diameter fibrils (mean diameter 70.04 ± 19.96 nm). Col. V formed much thinner fibrils (mean diameter 50.67 ± 5.63 nm). The Col. I fiber diameter is 88.75 ± 11.83 nm when the Col. V content is 10%. With the increasing amount of Col. V added, the diameter of collagen fibers first increases and then decreases. The Col. I fiber diameter is 60.21 ± 10.14 nm when the Col. V content is 70%.

**Figure 10.**
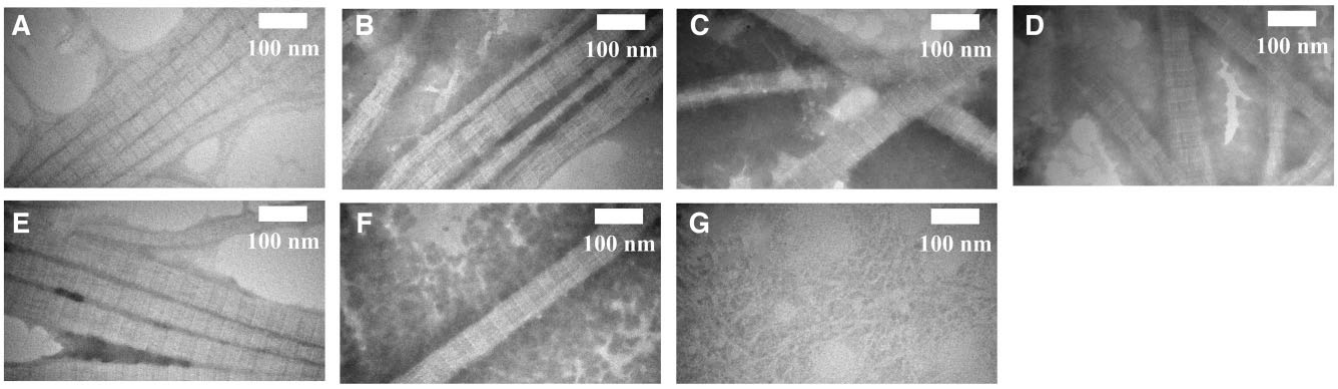
Co-assembly of Col. I and Col. V alters D periodicity. The total concentration of collagen is 50 µg/mL, and (**A–G**) is the amount of Col. V added (A: 0, B: 10%, C: 20%, D: 30%, E: 50%, F: 70%, G: 100%)

Figure 10 and Table 3 suggest that Col. I formed typical 61.42 ± 4.98 nm D-periodic while native Col. V formed considerably thinner fibrils without significant periodicity. With an increase in the amount of Col. V added, no changes were observed in the collagen D-periodic. Col. V cannot form the D-periodic, consistent with the results of other studies. This phenomenon might be induced by the removal of telopeptide from Col. V. The control is Col. V composition is 0% in Col. I.

**Table 3.** Co-assembly of Col. I and Col. V alters D periodicity

Col. V addition (%)	0	10	20	30	50	70	100
D periodicity (nm)	61.42±4.98	62.18±5.38	63.61±2.88	63.53±0.83	64.15±2.58	63.09±2.84	−

− indicates that D periodicity cannot be counted.

### Biocompatibility properties

#### Cell proliferation analysis

**Figure 11.**
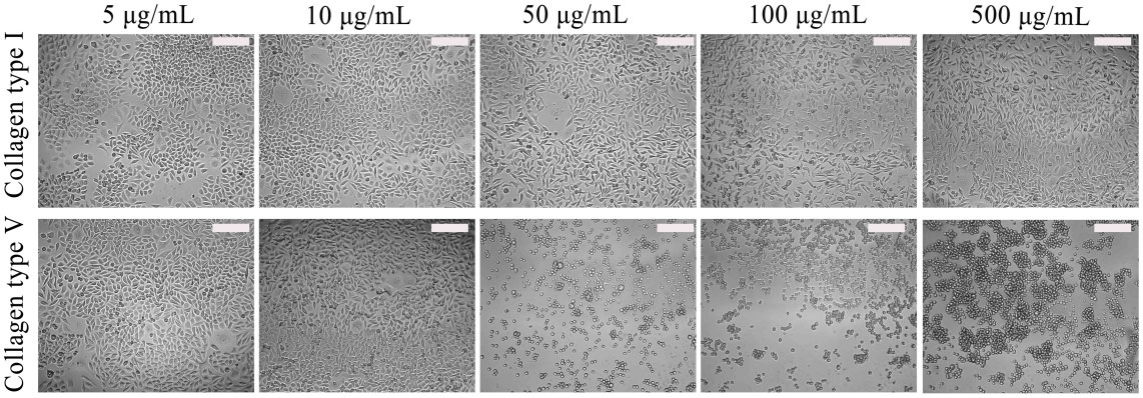
Effects of different concentrations of Col. I and Col. V on the growth of L929. Scale bar: 200 μm

In Fig. 11, Col. V presented different cell growth properties from Col. I. The same volume of cell suspension was added to each well during cell culture. The results revealed that the growth of L929 had no dramatic inhibitory effect as the concentration of Col. I increased. Meanwhile, no cell shrinkage or death was observed. In the low concentration range (5–10 µg/mL), the inhibitory effect of Col. V was not obvious. However, in the high concentration range (50–500 µg/mL), the growth of L929 was significantly inhibited with increasing Col. V concentration. The inhibitory effect appeared when the concentration of Col. V was 50 µg/mL. To further investigate the effects of the proportion of Col. V to Col. I on cell adhesion and proliferation behaviors, the concentration of 50 µg/mL was selected for cell culture, as this effects may be difficult to be observed at low concentration (5 µg/mL or 10 µg/mL) of Col. V.

The total concentration of collagen is 50 µg/mL.

**Figure 12.**
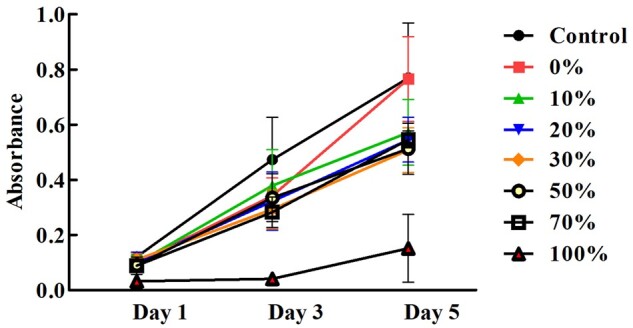
Cell proliferation curves of different Col. V additions

**Figure 13.**
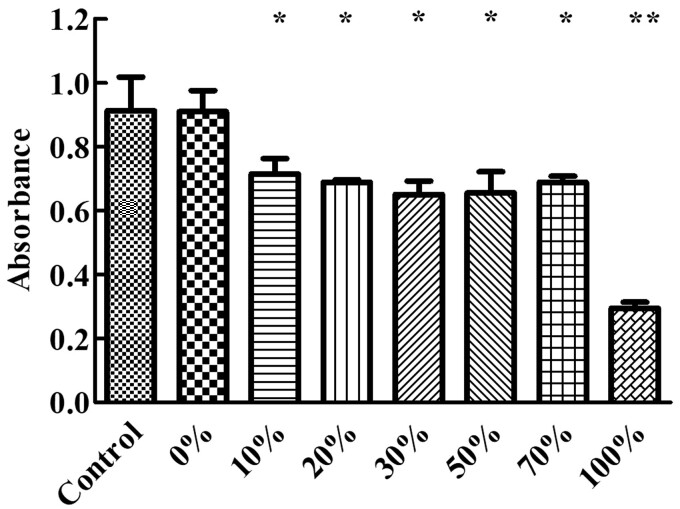
Histogram of cell absorbance value on the fifth day. The total concentration of collagen is 50 µg/mL. *P <0.05 and **P <0.01 vs. control

On Days 1 and 3 of Fig. 12, no significant difference on cell growth and in the absorbance value of different Col. V additions (0–70%) were observed, while the absorbance value of the control group was higher than that of the collagen supplemented group, and the group with 100% Col. V supplementation had the smallest absorbance value. On the fifth day (Fig. 13), except for the group added with 0% Col. V, the absorbance of the other groups was lower than that of the control group. There was no significant difference in absorbance when the Col. V was added at 10–70%. Nevertheless, the absorbance value dramatically decreased when the amount of Col. V added was 100%.

#### Cell scratch test analysis

**Figure 14.**
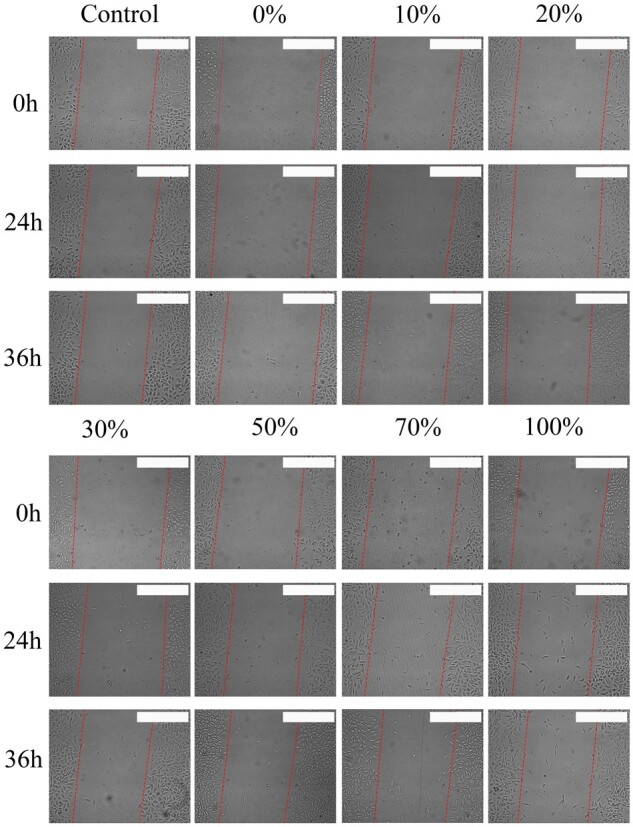
The scratch closure rate of cells with different proportion of Col. V was added. The total concentration of collagen is 50 µg/mL. Scale bar: 400 μm

**Figure 15.**
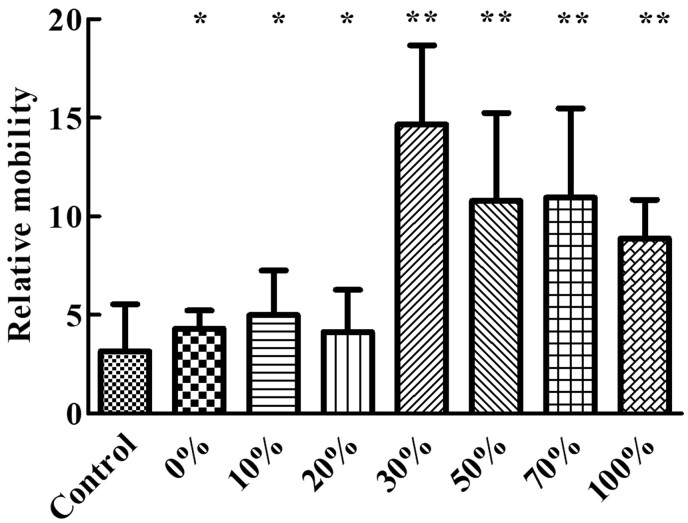
Histogram of scratch closure rate at 36 h. The total concentration of collagen is 50 µg/mL. *P<0.05 and **P <0.01 vs. control

Figure 14 shows the scratch closure rate of cells with different proportion of Col. V added from 0 h to 36 h. The scratch closure rate at 36 h is summarized in Fig. 15. It can be observed that compared with the control group, the addition of Col. V could promote cell migration. There was no significant difference in cell migration rates when 0–20% Col. V was added, and the cell migration rate dramatically increased and reached the highest when 30% Col. V was added. Although the cell migration was slightly inhibited when the added Col. V was in the range of 30–100%, addition of Col. V was shown to promote cell migration.

#### Confocal laser scanning microscopy observation

**Figure 16.**
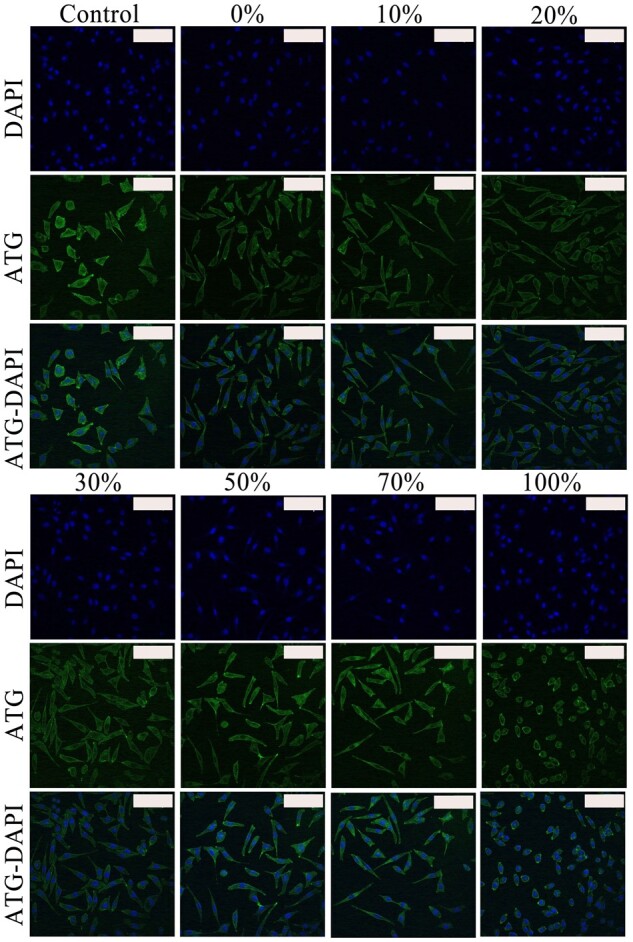
Representative confocal laser scanning microscopy images of L929 cultured with different amounts of Col. V were added. Scale bar: 200 μm

Figure 16 shows that the cells cultured on collagen-coated well had a spindle-shaped structure. Compared with the control group, the cells exhibited a spherical shape, indicating that the cell shape was associated with the collagen type and abundance. F-actin was stained with actin-tracker green (green), and the nucleus was stained with DAPI (blue). No significant change was observed in the size of the nucleus compared to the cytoplasm when the amount of Col. V was 0–70%. At this time, both presented a fusiform structure. Besides, the cell shrink and morphology became round when the ratio of Col. V reached 100% (Fig. 16).

## Discussions

Compared with the conventional method, the experimental group had higher Col. V content and less impurity protein content (Figs 1 and 2). This is mainly due to the introduction of ultrafiltration in the proposed method to further purify the Col. V, thereby removing the impurity proteins. Although the ultrafiltration is a widely reported method for protein purification [23], to the best of our knowledge, this technique has not been used for Col. V purification. According to SDS-PAEG results, the MW of collagen is > 100 kDa, while the MW of pepsin and other impurities is small. The ultrafiltration MW cut-off selected in the control group was 100 kDa, which could effectively remove impurity proteins, thus increasing the purity of Col. V. Col. V exists in various forms, such as [α_1_(**V**)]_2_[α_2_(**V**)], α_1_(**V**)α_2_(**V**) α_3_(**V**), [α_1_(**V**)]_3_ and hybrid Type **V**/XI [24] in different tissues. SDS-PAGE of Col. V suggested that the main composition of Col. V of the bovine cornea is [α_1_(**V**)]_2_[α_2_(**V**)]. The MW of Col. V was higher than that of Col. I, consistent with Silver’s report [25]. The obtained Col. V exhibited high purity and complete structure, implying that enzymatic hydrolysis coupled with membrane separation is effective for the separation of Col. V.

Col. V has a similar amino acid composition to Col. I. The contents of some amino acids are different. As suggested in Table 2, the Ala content in Col. I (112/1000 residues) was higher than that in Col. V (41/1000 residues). This is a typical difference between Col. I and Col. V [26]. Gly, Pro and Hyp are high abundance amino acids in collagen and play a key role in stabilizing the triple helix of collagen. The contents of Gly, Pro and Hyp in Col. V were higher compared to Col. I, contributing to the stability of Col. V being stronger than that of Col. I. The main hydrophobic amino acids in collagen are Leu, Ile, Phe and Met. In Col. V, the content of hydrophobic amino acids was slightly higher compared to Type I. The diameter of collagen fiber is associated with the content of hydrophobic amino acids. The higher hydrophobic amino acid may hinder the formation of thick collagen fibers. The diameter difference between Col. I and Col. V might be induced by the amino acid difference between collagen types (Figs 8 and 9). It was reported that Col. V can regulate the diameter fibers, but not the D-period of Col. I. In the cornea, Col. V plays a crucial role in regulating the initial fibril assembly [2, 27].

Birk investigated the effect of the addition of Col. V on fiber diameter [27] using only 10–30% of Col. V. This research expanded the ratio of Col. V and obtained more information on the role of Col. V in regulating fibers. Col. I and Col. V interact *in vitro* as heterotypic fibrils and have the same characteristics as those observed *in vivo* with respect to the masking of the Col. V helical epitopes. This interaction is partially responsible for the control of collagen fibril diameter. The NH_2_ domain of the Col. V molecule is required for the full effect. However, the removal of the NH_2_-terminal domain of the Col. V required high concentrations to produce a measurable decrease in the mean fibril diameter. Col. I formed fibrils diameters with a wide range (Figs 8 and 9). A D-periodic cross-striated pattern is a typical characteristic of collagen fibrils obtained by self-assembly and is essential for the mechanical and biological properties of collagen-based matrices [28]. The D-periodic arrangement is also critical for the formation of heterotypic structures consisting of fibrillar collagens and other collagenous or noncollagenous macromolecules [29]. This study revealed that Col. V can regulate the diameter of Col. I but not the D-periodic of Col. I (Fig. 8 and Table 3). This is consistent with the previous report [27]. This would be caused by the inconsistent conditions for forming collagen fibers, collagen type and/or concentration. The results demonstrate the potential biological effect of Col. V in tissue engineering.

Col. V selectively inhibits the growth of human umbilical vein endothelial cells, where Col. I, Col. III or Col. IV or other fibronectins enhance cell proliferation. The membrane glycoprotein complex Ia–IIa of platelet adheres to Col. I, Col. II, Col. III and Col. IV while it does not adhere to Col. V [30–32]. Col. V with high concentration can make L929 fall off the culture medium and gather into clusters (Fig. 11), similar to the Takanori’s result [33]. With an increase in the Col. V proportion, the diameter of collagen fibers was changed, which further affected the combination of collagen with cells.

Col. V may influence L929 from different aspects. Collagen type would influence the proliferation of L929 since collagens exhibit different combination properties with the cell surface. Col. I promotes the proliferation of L929, and Col. V presents inhibition properties [32]. Collagen types are associated with the MMP-1 enzyme activity and thus impact the proliferation of L929 [34]. Besides, Col. V controls the proliferation of L929 by changing the stiffness of the matrix [35]. Among three possible reasons, the dominant factors in regulating cell behaviors should be further investigated.

Scratch tests are commonly employed to estimate the wound healing rate *in vitro* [36, 37]. Scratch experiments with low serum concentration during culture may reflect the migration property of L929. Col. V with thinner fibrils may promote the migration of L929. Col. V can regulate the fiber diameter of Col. I (Fig. 9). With the addition of Col. V, the diameter of collagen fibers first increased and then decreased, presenting a relationship consistent with the migration (Fig. 15). Existing studies have shown that Col. I can promote the migration of fibroblast cells and has the potential to promote wound healing [36]. The results of this study showed that Col. I and Col. V together promoted cell migration better than Col. V alone. The addition of Col. V did not affect cells unilaterally, but was considered to be the results of comprehensive factors. As a result, there was no significant difference in cell migration when collagen V was added in the range of 30–100%.

L929 is commonly used for the evaluation of biomaterial’s effect on cell morphology adhesion and diffusion properties [38]. Collagen is frequently used for culture plate coating. In this experiment, the cell adhesion and migration were consistent with the arrangement of collagen (Figs 9 and 16). The filopodia- and lamellipodia-like extensions, as well as the elongated and spreading morphology, demonstrated excellent cellular growth behavior [39]. Compared with the control group, the collagen-coated plate revealed better L929 morphology (Fig. 16). The mobile cell membrane wrapped around the collagen when the L929 adhered to the small fiber-diameter coated cell plate, making the L929 membrane curvature larger and the conformation become a slender spindle-like figure. From another perspective, the multi-level of cells was observed when the cells adhered to the cell plate without collagen coating. Similar results were obtained by Dias *et al.* [40].

This paper discovered that Col. V can inhibit fibroblast proliferation while promoting migration. The effects of Col. V on other cells such as melanocytes should be evaluated to reveal more details of collagen properties on cell behaviors.

## Conclusions

Col. V is relatively abundant in the cornea and was used for the Col. V extraction. Col. V extracted by the proposed process exhibited a complete triple helix structure. The collagen fiber diameter was regulated by the addition of Col. V. The cell culture results suggested that Col. V inhibited and promoted the proliferation and migration of L929, respectively. Moreover, there was a maximum relative migration rate when the fiber diameter was 84.51 nm. However, Col. V has different characteristics from other collagens in cell–collagen interaction. Thus, more experiments should be conducted in the future to explore its effects on cell behavior.

## Funding

This work was supported by the National Key Technology R&D Programs of China (2021YFC2400800), Science and Technology Program of Guangzhou, China (201803010086), Open Funding Project of the State Key Laboratory of Biochemical Engineering (2021KF-04) and Independent Research Project of the State Key Laboratory of Biochemical Engineering (2021ZZ-03).

*Conflict of interest statement*. None declared.
